# Digital holographic microscopy for real-time observation of surface-relief grating formation on azobenzene-containing films

**DOI:** 10.1038/s41598-020-76573-6

**Published:** 2020-11-12

**Authors:** Heikki Rekola, Alex Berdin, Chiara Fedele, Matti Virkki, Arri Priimagi

**Affiliations:** 1 Smart Photonic Materials, Faculty of Engineering and Natural Sciences, Tampere University, P. O. Box 541, 33101 Tampere, Finland; 2 Department of Physics and Mathematics, University of Eastern Finland, P.O. Box 111, 80101 Joensuu, Finland

**Keywords:** Lithography, Interference microscopy

## Abstract

Light-induced surface structuring of azobenzene-containing films allows for creation of complex surface relief patterns with varying heights, patterns which would be difficult to create using conventional lithography tools. In order to realize the full potential of these patternable surfaces, understanding their formation dynamics and response to different types of light fields is crucial. In the present work we introduce digital holographic microscopy (DHM) for real time, in-situ observation of surface-relief grating (SRG) formation on azobenzene-containing films. This instrument allows us to measure the surface topography of films while illuminating them with two individually controlled laser beams for creating periodically varying patterns. By utilizing the information of the grating formation dynamics, we combine multiple grating patterns to create pixels with wide gamut structural colors as well as blazed grating structures on the film surface. As long as the material behaviour is linear, any Fourier optical surface can be created utilizing this multiple patterning approach. The DHM instrument presented here has the potential for creating complex 3D surface reliefs with nanometric precision.

## Introduction

Active control over material properties such as colour, dielectric permittivity, refractive index, and surface shapes drives modern visual technology^1^. By utilizing light-active molecules, such as photochromic molecules that can reversibly photoswitch between two or more conformations^2,3^, the active control can be remotely driven with light. This property allows wireless access to such switchable systems, whose shape can be precisely programmed dynamically and locally. One particularly intriguing phenomenon observed with azobenzene-based photochromic materials^4^ is the light-induced mass migration^5,6^, which can be used to create topographical patterns on thin films in response to patterned illumination in a reversible way^7^. Such light-reconfigurable topographies are of great interest in e.g. biology^8^, wettability control^9^, and tunable elastic metamaterials^10^, all the way to the microfabrication of photonic elements^11,12^.

The light-induced topographical patterns on azomaterial surfaces have found applications in visualizing the near-fields of plasmonic structures^13,14^ and the orbital angular momentum of a light beam^15^. For lithography, both arbitrary^16^ and periodic^17^ patterns can be created using interference lithography. The so-called surface relief gratings (SRGs) can be created from two or more laser beam interference patterns, or by consecutive exposures to rotated interference patterns, resulting in periodic modulation of the surface topography from one or two dimensional gratings to quasicrystal structures^18^. Even more complex patterns can be obtained using computer-generated holograms^16^, or by fully exploiting the polarization-dependent response of the azomaterials^19^.

Besides the many different application fields where azomaterials are considered extremely promising, the basic understanding and direct observation of mass migration in these materials still holds great interest. The periodic intensity or polarization direction patterns formed by interfering laser beams serve as convenient test patterns for assessing the material behaviour^3,20^. In-situ measurements of the grating development are typically done by monitoring the diffraction efficiency^21–23^, which gives an averaged estimate of the grating shape over the probed area. More details can be accessed with in-situ scanning probe methods, either with atomic force microscopy (AFM)^20,23,24^ or with near-field optical microscopy (NSOM)^25–27^ techniques. Such experiments require a customized instrument combining the scanning probe and the illumination setups. Typically, a line scan is performed repeatedly as the grating is forming, giving information about the changes on the surface of the film. In order to directly observe fast dynamics over a wider area, a non-scanning, non-contact measurement technique would be beneficial. This would also enable observations of a 2D area instead of a single line, potentially giving additional information.

Digital holographic microscopy (DHM) is a quantitative phase imaging technique^28–30^, where both the amplitude and phase of light interacting with a sample are measured. The phase contains information on the height and refractive index variations on a sample. In the off-axis configuration, the hologram is created between light from a sample and a reference wavefront hitting a sensor at an angle, allowing the separation of the direct reflections and the interference pattern in the frequency domain^29,31^. This permits the observation of fast dynamics for a full 2D area, as the phase of the reflected/transmitted light can be retrieved numerically from a single hologram. Thus, the update rate in the surface topography and refractive index profile of a sample is limited only by the frame rate of the camera used to record the holograms. Pagliarulo et al.^32^ have shown how this technique can be used in transmission mode with a conventional Lloyd-type interference lithography setup to monitor the grating formation on an azobenzene functionalised polymer.

Here we introduce a new instrument for studying surface relief grating inscription, a commercial DHM operating in reflection where we have integrated an interference lithography system. The incident angles, polarizations, and the relative phase difference of the two laser beams used for creating the interference patterns can be chosen independently. The dynamics of the grating formation can be recorded with the full 2D field of view with a time step of 5 ms between measurements. The sample position can be scanned using computer control, allowing for patterning selected areas with grating structures. This enables us to pattern full color images by overlaying gratings that scatter red, green, and blue colors with desired intensities on top of each other that defines one pixel of the image. Finally, we demonstrate formation of a blazed grating by sequential patterning of a fundamental grating and its first three harmonics with a precisely controlled phase offset between the four patterns.

## Results and discussion

### DHM observation principle and interference lithography

**Figure 1:**
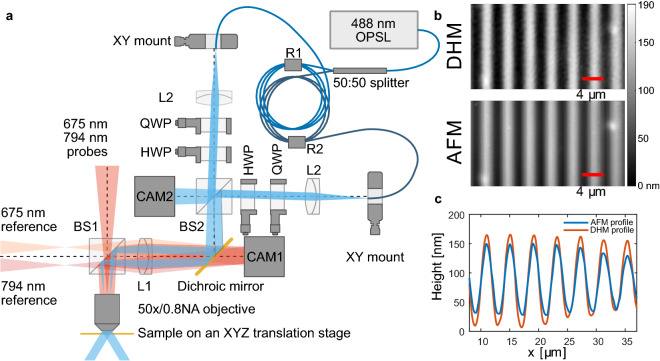
Optical measurement setup for simultaneous surface height measurement and SRG patterning. (**a**) Schematic of the optical components used to project the interference pattern for SRG writing through the digital holographic microscope. The light input (488 nm) to the microscope is through a polarization maintaining fibre, output of which is collimated by lenses L2. After adjusting the polarization states with quarter- and half-wave plates (QWP and HWP, respectively), the two beams are combined with a 50:50 beam splitter BS2. The combined beam is then focused onto the back focal plane of the microscope objective by lens L1, resulting in two collimated beams exiting the objective. The angles of these two beams are controlled by the XY mounts for the ends of the optical fibre. (**b**) DHM and AFM images of an SRG with $4\ \upmu \hbox {m}$ period inscribed with the optical setup shown in (**a**) using left- and right-handed circular polarizations for the inscription. The grating cross sections are compared in (**c**), with the DHM measurement shown in orange color and the AFM measurement in blue.

We use a commercially available digital holographic microscope (DHM R-2100, LyncéeTec), that we have modified with the capability of projecting two collimated 488 nm laser beams through the microscope objective with freely selectable angle and polarization state. The setup and the outline of the components used are shown in Fig. 1a. As both the surface height measurement and the interference pattern projection are done through the microscope objective, the sample can be freely moved on an XYZ-translation stage (Prior ProScan HT1111, Prior FB205E). As samples we use regular microscope glass slides coated with a $480 \pm 20\ \hbox {nm}$ thin film of Disperse Red 1 glass (DR1g, Solaris Chem Inc., Canada), which known to be an efficient SRG forming material^33^. The films were created by spin coating a of 9% (w/v) solution in chloroform at 1500 rpm for 30 s.

The DHM uses two diode laser sources with wavelengths of 675 nm and 794 nm (shown with red color in Fig. 1a) to both probe the reflection from a sample and as a reference beam for the interferometric observation. The two beams pass through a 50:50 beamsplitter (BS1 in Fig. 1a), with the probe beam focused onto the back focal plane (BFP) of an objective (50 $\times$, 0.8NA, Olympus MPLFLN50X), resulting in a plane-wave-like illumination of the sample. The reflected light from the sample is collected by the same objective, and imaged through the beamsplitter near the camera CAM1 (Basler acA1920-155um) in Fig. 1a by the lens L1. The reference beam is collimated by the same lens L1, resulting in an interference pattern on the CAM1, i.e. the hologram, which is then used for the reconstruction of the phase and intensity information of the light reflected from the sample. The image of the sample is placed intentionally slightly out of focus on the CAM1, and the correct focus is recovered numerically during the reconstruction process^28^.

An example of a grating produced using the DHM with $4\ \upmu \hbox {m}$ period with circularly polarized writing beams is given in Fig. 1b. The grating profile is imaged with both the DHM and an AFM. The cross sections of the grating profile are compared in Fig. 1c. The two different techniques show the same sinusoidal shape for the grating profile, but disagree slightly on the height of the grating with the DHM overestimating the height. We will return to the possible causes of this discrepancy when discussing the image reconstruction algorithm used in the DHM. The AFM measurements were conducted using a Park XE-100 AFM (Park Systems corp., Korea) in non-contact mode (in air). We use an Al-coated Si ACTA probe (Applied NanoStructures Inc., California, USA) with a nominal frequency of 200–400 kHz, a spring constant in the range 13–77 N/m, with a tip radius below 10 nm.

The beam path and components used for the interference pattern creation are shown with blue color in Fig. 1a. We use a 488 nm optically pumped semiconductor laser (Coherent Genesis CX-488 2000) that is coupled into a one-to-two beam splitting, polarization maintaining, single mode optical fibre (Thorlabs PN480R5F1). The split ratio is nominally 50:50, and the output from each fibre end is collimated with a 40 mm focal length triplet lens L2 (Thorlabs TRH254-040-A-ML), followed by a quarter wave plate (QWP, Newport 10RP04-12), and a half wave plate (HWP, Newport 10RP02-12) to control the polarization state of the beams. These two collimated beams are combined using a 50:50 beam splitter BS2 (Thorlabs BS013). The combined beam is then coupled to the optical path of the DHM with a dichroic mirror (Semrock Di03-R488-t3). Finally, the combined beam entering the microscope is transformed into two points on the back focal plane of the objective by the lens L1 (Olympus SWTLU-C, 180 mm focal length), with each point corresponding to the image of an optical fibre end created by the lenses L2 and L1. The objective converts the two points on the back focal plane to two collimated beams exiting the objective.

The propagation direction of the two beams exiting the objective depends on the location of the two points projected to the back focal plane. These locations are controlled by the location of the fibre ends, which are mounted on XY-translation stages. Initially we center the fibre end positions such that when the sample stage of the microscope is moved down, no lateral movement is observed for the laser spot on the sample. Then one of the fibres is moved to generate an interference pattern with double the desired period, and finally the second fibre end is moved to get the target period for the pattern. This ensures that the writing beams are symmetric with respect to the optical axis. The maximum angle between the beam and the sample normal is limited by the numerical aperture of the objective, which in our case is $\sin ^{-1}(0.8) \approx 53^{\circ }$. The minimum period for the interference pattern is then given by $\Lambda = 488/(2 \times 0.8) = 305\ \hbox {nm}$. This is below the spatial resolution of the DHM, and in order to observe the SRG formation it is necessary to collect at least the first diffracted orders from the grating at the DHM probe wavelengths, which corresponds to a grating period of approximately $1\ \upmu \hbox {m}$.

The polarization states for the two writing beams are controlled by the positions of the HWP and QWP on each beam path. The different polarization states were calibrated by placing an orthogonal polarizer and a photodiode as the sample, and minimizing the signal on the photodiode by adjusting the HWP and QWP angles. The camera CAM2 (Flir BFS-U3-12S4M-CS) is used for observing the interference pattern that is projected to the sample. The pattern seen on CAM2 is 50 × larger compared to the pattern projected on the sample. This allows for accurate adjustment of the grating period and the illumination intensity on the sample. A manual shutter below the beamsplitter BS2 in Fig. 1a can be used to block the 488 nm laser from entering the microscope while adjusting the grating parameters. The maximum illumination intensity available at the sample is $4\ \hbox {W}/\hbox {cm}^2$, limited by the power handling of the optical fibre.

**Figure 2:**
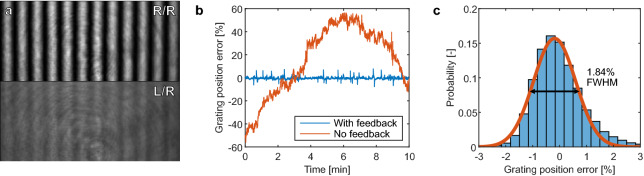
(**a**) Feedback camera (CAM2) images for intensity (top) and polarization (bottom) interference patterns. The resulting grating period on the sample plane is $4\ \upmu \hbox {m}$. The polarization interference pattern becomes visible due to a slight imbalance in the laser power of the two interfering beams, enabling the active feedback based on the camera signal for both types of patterns. (**b**) Relative error (compared to the grating period) in the grating position over time with (blue) and without (orange) active feedback enabled. (**c**) Histogram for the error in the grating positioning over 27 min with the active feedback enabled.

Finally, a key component in the function of the interference pattern generation is an active feedback loop for controlling the phase difference of the two interfering beams. The two optical fibres after the 50:50 splitter are sensitive to minute changes in temperature, leading to variance in the optical path length between the two arms of the interferometer. These changes in the relative phase between the two beams shift the location of the interference pattern on the sample. In order to minimize the thermal effects, the fibre 50:50 splitter and the optical fibres following it are thermally insulated. In addition, two $220\, \Omega$ resistors are connected to the fibre loops inside the insulation, one for each arm of the interferometer, shown as R1 and R2 in Fig. 1a. Applying a voltage to these resistors allows us to utilize the thermal sensitivity of the optical fibre to actively control the phase difference between the two interferometer arms. The feedback loop is controlled by a Python program that reads the images from CAM2 (Fig. 2a top panel) and calculates the current grating pattern parameters by fitting the function

1 $$\begin{aligned} I(x,y) = A\cdot \cos {\left( \frac{2\pi }{p}(x\cdot \cos (\alpha ) +y\cdot \sin (\alpha ))+\delta \right) }+ B \end{aligned}$$

where *A* is the grating amplitude, *B* is the background signal level, *p* the grating period, $\alpha$ the grating orientation angle and $\delta$ a phase term describing the location of the grating maxima. The deviation of the phase term $\delta$ from a setpoint value is used as the error signal for a Proportional–Integral–Derivative (PID) control loop, which is then finally translated into an output voltage using an Arduino microcontroller directly attached to the resistors R1 and R2. Without the active feedback, the grating position drifts randomly over time, as shown in Fig. 2b. With the active feedback enabled, the relative error in the grating position is stabilized to below a few percent (Fig. 2c). The feedback signal is based on the intensity patterns observed on CAM2. However it can be used also for polarization interference patterns, as the two arms of the interferometer have a $2\%$ difference in power due to the beamsplitters used, resulting in a pattern to monitor on CAM2 (Fig. 2a bottom panel).

### DHM for monitoring SRG inscription dynamics

**Figure 3:**
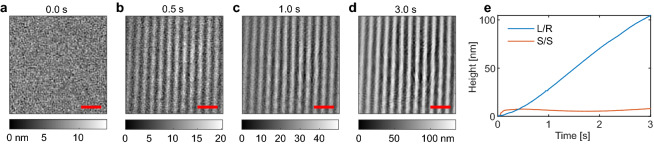
SRG inscription as captured by the DHM. In (**a**–**d**) 2D height maps of a $4\ \upmu \hbox {m}$ period grating written utilizing left- and right-handed circular polarizations for the writing beams. The red scale bars in the figures correspond to a $10\ \upmu \hbox {m}$ distance. The 2D height maps are captured at a 200 fps frame rate, with single measurements at 0.0 s, 0.5 s, 1.0 s, and 3.0 s after opening the shutter for the writing beams shown in (**a**–**d**), respectively. In (**e**) the grating heights are shown for L/R and S/S polarizations for the writing beams with $2\ \hbox {W}/\hbox {cm}^2$ average intensity, showing fast SRG inscription for the L/R polarization, but only minor surface deformation for the S/S polarization combination, as expected.

To demonstrate the grating growth on a DR1g film utilizing the DHM, we inscribe two $4\ \upmu \hbox {m}$ period gratings, one with left- and right-handed circular polarizations (L/R) for the writing beams, and one with S-polarization for both beams (S/S). The L/R polarization combination is known to be an efficient choice with DR1g^33^, while the S/S polarization is not. The 2D profiles of the grating formed with L/R polarization is shown in Fig. 3a–d, where snapshots at 0.0 s, 0.5 s, 1.0 s, and 3.0 s after starting the inscription are shown (the full Video 1 is available as Supplementary Material). The inscription is done at a high intensity of $2\ \hbox {W}/\hbox {cm}^2$, which explains the rapid growth of the grating to over 100 nm average height in just 3 s. Lower intensities slow down the grating formation, but show qualitatively similar behavior. To obtain the average grating height, we fit Eq. (1) to the height maps obtained with the DHM using the 794 nm probe wavelength. The height is given by twice the amplitude value for the cosine function, i.e. 2*A*, and this is shown in Fig. 3e as a function of time for both writing beam polarization combinations. The L/R polarization shows that the grating growth rate initially increases, then continues linearly until the rate starts to slow down slightly after two seconds of inscription. With the S/S polarization however the grating height jumps to 6 nm, and stays nearly constant (see Supplementary Fig. S2 for AFM comparison). We believe this apparent minor surface modulation to be due to how the height maps are reconstructed from the phase of the reflected light in the DHM^29^.

The image reconstruction algorithm used in calculating the height maps in the DHM assumes that the observed phase differences between different points on the sample surface are solely due to differences in the optical path length between the objective and the sample surface. The sample material is assumed to have a uniform refractive index. These assumptions are not entirely valid for transparent films, as the reflection from the substrate can contribute to the measured phase differences. This is also the reason behind the discrepancy between the AFM and DHM height maps shown in Fig. 1b. By modelling the light propagation inside the material, the DHM analysis can be extended for measuring film thicknesses for homogeneous materials and to improve the accuracy of the height measurements of transparent films^34^.

**Figure 4:**
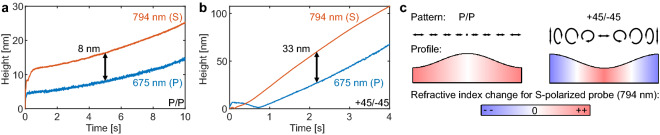
Effects from phase grating formation in DR1g films on the observed grating heights. In (**a**) the grating is written with P-polarized amplitude interference pattern, while in (**b**) a polarization interference pattern created with + 45/− 45 linearly polarized beams is used. The 675 nm and 794 nm labels refer to the probe wavelength used in the DHM, with the probe polarizations being close to P and S polarizations for 675 nm and 794 nm, respectively. The polarization directions for the interfering beams are depicted with arrows in (**c**), with the length of the lines illustrating the intensity distribution for the P/P pattern. The resulting grating profiles are shown schematically, with the color corresponding to the change in the refractive index due to the reorientation of the azobenzene molecules inside the film for S-polarized probe beam. For a P-polarized probe beam the change in the refractive index is in the opposite direction, i.e. the color scale is reversed.

For monitoring the grating growth dynamics the simpler reconstruction algorithm can cause inaccurate results. During the grating inscription, the azobenzene molecules orient perpendicular to the incident electric field^23^, resulting in bulk birefringent gratings in addition to the SRG formation. The contribution from the two grating types has been previously studied by analyzing the polarization state^21,35^ and the shape^23^ of beams diffracted by the gratings. The birefringent gratings can contribute in two ways to the DHM measurement. First, if the refractive index of the material has an imaginary part at the DHM probe wavelengths, then the phase of the light reflected from the air interface of the material will change as the molecules inside the film reorient. For the DR1g material used in here this is unlikely, as the absorbance of the material is close to zero at wavelengths larger than 630 nm^33^. The second contribution comes from light that is reflected from the substrate interface. Depending on the film thickness used, this reflection can add constructive or destructive interference to the main reflection from the top surface of the film, offsetting the measured grating height. The grating pattern seen with the S/S polarizations in Fig. 3e could be due to the birefringent grating forming in the bulk of the film, instead of a surface modulation.

To test how large of an effect the birefringent gratings have on the observed grating amplitudes we recorded the beginning of the SRG formation using two different polarization combinations for the writing beams. The two probe lasers in our DHM have different linear polarizations, with the 675 nm having an angle of − 9 degrees with respect to the x-axis, and the 794 nm having an angle of 72 degrees. With the grating lines along the y-axis, the 675 nm probe is closer to P-polarization, and the 794 nm to S-polarization. By comparing the observed SRG height, obtained by fitting Eq. (1) to the recorded height maps for the two probe wavelengths separately, we are able to estimate the uncertainty in the grating height as the effect of the birefringent grating should be positive for one probe wavelength, and negative for the other. Figure 4a,b show the observed SRG heights for the two probe wavelengths for two different writing beam polarizations. The average intensity for the writing beams was $2\, {\hbox {W}}/\hbox {cm}^2$, the periodicity $4\ \upmu \hbox {m}$, and the acquisition rate was set to 200 fps on the DHM.

With P/P polarized writing beams (Fig. 4c), where the electric field is linearly polarized perpendicular to the grating lines, the birefringence will be strongest at the intensity maxima, and at the intensity minima there is either weak or no birefringence. The intensity pattern is shown schematically with the arrows corresponding to the electric field direction over one period of the grating, with the length of the arrows describing the intensity of light. The resulting profile and refractive index distribution for S-polarized (along the grating lines) probe beam are shown below the field patterns. For a P-polarized probe, the change in the refractive index is in the opposite direction. In Fig. 4a we find that as soon as the writing beams are switched on, the observed SRG height jumps to 5–8 nm, from there the difference between the two probe wavelengths grows to 8 nm and stays approximately constant. In Fig. 4b a much larger difference is seen between the two probe wavelengths. Here, the polarization interference pattern created with linearly polarized writing beams at + 45 ° and − 45 ° orientations maximizes the contrast in the birefringent grating. Over one half-period, the molecular orientation switches from along the grating lines to perpendicular to the grating lines. The difference between the two probe wavelengths becomes 33 nm already within 2 s of inscription. The initial dynamics shows interesting behavior for the 675 nm probe wavelength, which is polarized perpendicular to the grating lines. Initially, as soon as the SRG writing is started, the observed height jumps to 8 nm as the birefringent grating forms. Then as the SRG grows in amplitude, the observed height initially decreases before starting to rise again as the SRG becomes the dominant signal.

In principle, extending the DHM to use two probes at 675 nm with orthogonal polarizations, and two probes at 794 nm with orthogonal polarizations would give enough information to reconstruct both the film thicknesses^34^ and the birefringent refractive index of the DR1g film as a function of the position^36,37^. This extensions would make the DHM an even more powerful and accurate tool for measuring the movement and dynamics of surfaces and thin films, where birefringence can be expected due to molecular ordering either in response to external stimulus, or due to stresses induced by the movement^38,39^.

In addition to the realization of new tools for the study of SRG dynamics, the features of the DHM-based SRG writing setup enable some interesting possibilities for writing special gratings that are demonstrated in the following sections. These features include (1) simple and precise tuning of the grating pitch, (2) adjustable phase locking of the interference pattern, (3) sub-mm size of the grating area and (4) ability to write multiple gratings on top of each other. Our first example is the writing of full color images which relies on features (1) and (3). We convert the color information of each pixel in an example image to the grating pitch of three primary colors and the grating heights required to reproduce the color by diffraction of white light. The second example relies on features (1) and (2) and we demonstrate the realization of a blazed diffraction grating as a combination of four Fourier components represented by phase-locked interference patterns with harmonic pitch relation to one another. Both examples require also the fourth feature which is inherent to light-induced SRGs written to azopolymers. While both of these examples rely on one-dimensional SRGs, the setup allows also for patterning two-dimensional SRGs (see Supplementary Fig. S3).

### True color diffractive pixels by multi-exposure SRGs

**Figure 5:**
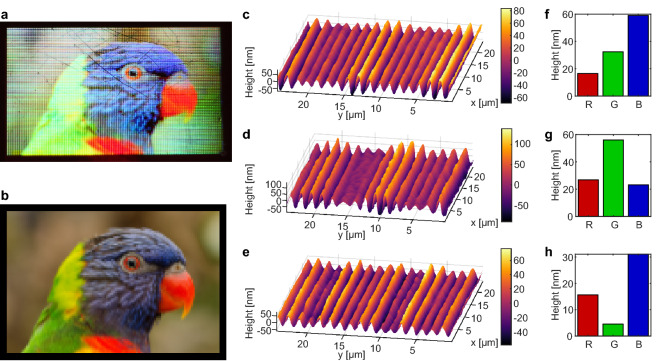
Full color image writing by stacking gratings. (**a**) A photograph of a Rainbow Lorikeet written on a DR1g film surface with 120 $\times$ 80 pixels. The total size of the written area is 12 mm $\times$ 8 mm, and the writing time was 9 h. The image was taken in direct sunlight, by capturing the first reflected diffraction order with a camera. Each pixel of the image contains three gratings for reflecting red, green, and blue light towards the camera. (**b**) Source image for patterning. Original photograph by Peter Heeling, licensed under the CC0 license^40^. (**c**–**e**) Surface profiles of three randomly selected pixels imaged with the DHM, consisting of a mixture of three grating periods with the measured grating heights shown in (**f**–**g**).

Structural colors have become an alternative to dyes and pigments for creating fade-free colored surfaces. Examples in the literature range from subwavelength plasmonic resonators, where a metallic nanostructure is created to have strong interaction with specific wavelengths of light^41^. These nanostructures push the resolution limits of color printing down to the diffraction limit of optics, albeit with a limited palette. More vibrant colors can be obtained from periodic photonic structures, many of which take inspiration from structures occurring in nature^42^. In the most basic form, the SRGs discussed here can be considered a method for creating structural colors.

Our goal was to translate a digital color image into SRG structures. Similar to the work on multi-exposure SRGs used for creating quasicrystal structures^18,43^, we created pixel structures by exposing a small area with three SRGs with different periods. Computer graphics programs often use 24-bit RGB coding for digital images. This means that the information for each pixel is stored in three channels with 8 bits each for red, green and blue. We used the interference lithography system of our DHM and the motorized sample stage to translate these pixel values into SRG structures. Three gratings were written on top of each other with the periods 1890 nm (3 $\times$ 630 nm) for red, 1596 nm (3 $\times$ 532 nm) for green and 1401 nm (3 $\times$ 467 nm) for blue pixels.

The intensity in each channel was translated into an exposure time for the SRG. Longer exposure times lead to higher grating height and thus to a higher diffraction efficiency. The dependency between exposure time and diffraction efficiency is linear for short exposure times of a few seconds^44^. By adjusting the exposure time for the three grating periods depending on the value of the three color channels of the pixel, the color from the digital image was reproduced in the diffracted light. The exposure time was set to 3 s for the maximum value 255 and 0 s for values below 30. These settings resulted in a maximum grating height of 150 nm, which had an excellent diffraction efficiency, but was still fast enough for writing larger holograms.

For larger scale holograms consisting of several thousand pixels, the translation stage was programmed with LabView to scan the sample three times, once for each channel with the exposure time corresponding to the channel intensity in the sample image. The field of view of the objective defines the size of the pixel to approximately $100\ \upmu \hbox {m}$. Therefore, the step size of the translation stage was also chosen to be $100\ \upmu \hbox {m}$ to avoid overlapping. This corresponds to a pixel density of 254 ppi, similar to a typical tablet display^45^. When sending a command to the translation stage, a delay of about $300\ \upmu \hbox {s}$ was observed before the stage started moving, which caused over-saturation for the darker color tones. Therefore, the pixels with intensity values below 30 were skipped in the writing process to minimize the impact of this lag.

Figure 5a shows a picture of a Rainbow lorikeet written on a DR1g film as stacked gratings. The source image is shown in Fig. 5b, and examples of the grating structures from three randomly selected pixels are shown in Fig. 5c–e (diffraction efficiency measurement is available as Supplementary Material). A beating pattern can be observed in the grating heights, corresponding to three overlapping periodic structures. The heights of the grating components in these example pixels are shown in Fig. 5f–h, calculated by taking the Fourier transform of the measured grating profiles.

### A Fourier-synthesized blazed grating

Interference of two plane-wave-like light beams creates a one-dimensional sinusoidal pattern that is replicated onto an azomaterial film. For a specific polarization combination, there are only two simple ways to modify the created pattern: adjustment of the angle between the two beams and the exposure dose. The former changes the pattern period and the latter the pattern amplitude while the created profile is typically sinusoidal. Our approach with precise control of pitch, direction and position (phase) of the sinusoidal pattern allows an arbitrary one- or two-dimensional pattern to be created with multiple exposures by taking advantage of Fourier analysis.

The foundation of Fourier analysis is that an arbitrary repeating pattern can be created by summing harmonically related sine and cosine functions. However, this requires that the harmonic relation between the components is very accurate, i.e., the period of each function needs to be that of the longest period component divided by an integer. Any deviation quickly leads to distortion of the summed function form over several periods. In addition, the pattern placement must be highly accurate, within a fraction of the period of the sines and cosines to be summed. Our approach with camera-determined period and active phase locking covers both requirements and enables using the Fourier analysis approach to SRGs. This allows for the ability to create any one-dimensional repeating pattern, and combined with pattern rotation, any two-dimensional repeating pattern. Similar approach has been used to create blazed gratings in photoresists using two Fourier components^46,47^.

**Figure 6:**
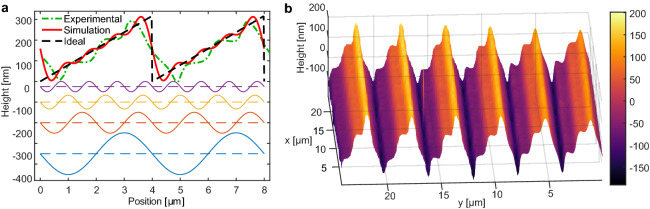
(**a**) The ideal, simulated four-sine target and realized profile for a blazed grating. The Fourier components are offset to the negative range. (**b**) The realized grating profile as measured by the DHM. The difference between the simulation and the experiment comes mainly from the second and third harmonic components of the grating being smaller than intended in height, but overall the blazed nature of the realized grating is clearly visible.

As an example, we created a sawtooth profile applicable as a blazed diffraction grating. Fourier series of a sawtooth profile (Fig. 6a) is composed of a constant term which relates to sample thickness and sines with decreasing amplitude. We chose to limit to the four lowest spatial frequencies meaning the sinusoidal patterns with largest pitches and selected $4\ \upmu \hbox {m}$ for the largest pitch. Therefore, we exposed the same sample spot with interference patterns with pitches of 4, 2, 1.33, and $1\ \upmu \hbox {m}$ (fundamental period divided by 1, 2, 3, and 4 respectively), utilizing the active feedback to expose each grating in the same place and with the same phase on the sample. In order to get more uniform grating structures, the grating directions are aligned with the sample stage movement direction, and the stage is scanned back and forth while exposing the sample. This averages out the intensity distribution of the writing beam in the movement direction, resulting in a more consistent grating profile (Fig. 6b). The target sawtooth profile with 4.54 degree angle blaze angle and $4\ \upmu \hbox {m}$ pitch corresponds to a blazed grating in the Littrow configuration optimized for 633 nm wavelength.

The ideal sawtooth profile, simulated profile as the sum of four sines, and profile of the realized grating are shown in Fig. 6a. The form and amplitude of the written profile matches the simulated with reasonable accuracy. The realized (simulated) gratings had heights of 198.3 nm (202.2 nm), 39.9 nm (101.1 nm), 15.2 nm (67.4 nm), and 28.98 nm (50.45 nm) for the $4\ \upmu \hbox {m}$, $2\ \upmu \hbox {m}$, $1.33\ \upmu \hbox {m}$, and $1\ \upmu \hbox {m}$ components, respectively. The smaller than intended amplitudes for the second and third harmonics especially explains the slight differences between the obtained and simulated grating profiles. In the experiment, the writing times for the different components were predetermined from separate measurements on a flat film. Ideally, the DHM measurement would be used as feedback to adjust the exposure times for more accurate patterning, but for this first demonstration this was not done. The consistency of the grating profile was excellent even over larger distances, thanks to the scanning movement of the stage averaging out errors in the interference patterns.

## Conclusions

For studying SRG formation using interference lithography, the freedom to choose the parameters for the interference pattern are basic requirements. Having the possibility of monitoring the SRG formation in-situ, and being able to control the pattern location precisely are features that enable new possibilities for applications such as creating Fourier-optical surfaces or printing structural colors in azomaterial films. These examples were enabled by the unique combination of being able to iterate fast thanks to the immediate feedback provided by the combination of the DHM instrument and the azobenzene material, as no additional process steps were needed to see the resulting patterns.

The DHM instrument introduced here enables the observation of the SRG formation with high spatial and temporal resolution, and the active feedback for the interference lithography addition enables control over the phase difference between the two interfering beams. The grating heights measured with the DHM deviate slightly from those recorded with AFM, and the birefringence induced in the material can cause an additional deviation in the recorded height. These are issues that can be solved by better accounting for the material properties in the hologram reconstruction, which would at the same time provide spatially resolved information of the SRG and the birefringent gratings separately.

Based on these early results, the DHM instrument is an extremely powerful tool that enables observing the SRG formation with a level of detail previously inaccessible. We think that it will add significant value to the studies on birefringence and SRG formation on any photochromic materials. For surface patterning, the real-time feedback, once fully integrated into the pattern generation would enable fabrication of arbitrary 3D surface profiles. This is something that is either very difficult to do with conventional lithography tools, or a very slow process of writing and measuring the surface point by point with thermal scanning probe microscopy^48^.

## Supplementary information

Supplementary Information.

Supplementary Video 1.
