# Supplementary material for: Digital holographic microscopy for real-time observation of surface-relief grating formation on azobenzene-containing films
Source: Sci Rep. 2020 Nov 12;10:19642. doi: 10.1038/s41598-020-76573-6 (PMC7665031; doi:10.1038/s41598-020-76573-6)
Supplement: Supplementary file 1 — Supplementary Information. [file 41598_2020_76573_MOESM1_ESM.pdf]

Digital holographic microscopy for real-time observation of surface-relief grating formation on azobenzene-containing films

Heikki Rekola[1,2], Alex Berdin[1], Chiara Fedele[1], Matti Virkki[1], and Arri Priimagi[1,*]

[1]Smart Photonic Materials, Faculty of Engineering and Natural Sciences, Tampere University, P. O. Box 541, FI-33101 Tampere, Finland
[2]Department of Mathematics and Physics, University of Eastern Finland, P.O. Box 111, FI-80101, Joensuu, Finland.
[*]arri.priimagi@tuni.fi

Supplementary Information

Diffraction efficiency measurements

The diffraction efficiency measurements were performed on seven gratings to diffract the three primary colors red, green and blue, and their additive combinations, yellow, magenta, cyan and white, at the same angle. These gratings consist of pixels similar to the Figure 5 of the main manuscript, patterned to cover a 1 mm^2 area for each color. The probe beam was a monochromatic light source (CPS670F, Thorlabs) emitting at a wavelength of 670 nm. The sample was adjusted to an angle of about 20 degrees with respect to the incident beam. A power meter (LabMax T0, Coherent) with the detector OP-2 VIS (Coherent) was used to measure the amount of light in the first transmitted diffraction order for each constituent grating period for the differently coloured pixels.

As each of the three grating components is designed to diffract a certain wavelength of light (630/532/467 nm) into the same angle with respect to white coming towards the sample, the expected result for the case of narrow band illumination to a "white" pixel is three diffraction maxima closely spaced to each other for all visible diffraction orders. Similarly only one diffraction spot should appear for a "red", "green" or "blue" pixel and two for "yellow", "cyan" and "magenta". This behavior is seen in Figure S1 where the first diffraction order created by such colored pixels is shown.

We observe a lower diffraction efficiency (Table S1) for the red and green component compared to the blue component, although the gratings had the same exposure time during the holographic inscription. This leads to the conclusion that gratings with higher periods experience slower amplitude growth compared to gratings with shorter periods. In future work the inscription time for the components must be calibrated with respect to the diffraction efficiency.

Figure S1 (b)-(c) shows the diffraction pattern of all 7 gratings probed with a expanded probe beam. The diffraction pattern represents the combination of the designed grating periods (Figure S1 (b)), which were superposed during the inscription process.

Color combination	Red efficiency	Green efficiency	Blue efficiency	Red height	Green height	Blue height
Red	3.3%	0%	0%	64 nm	2 nm	0 nm
Green	0%	5.4%	0%	1 nm	98 nm	1 nm
Yellow	1.3%	3.5%	0%	20 nm	55 nm	5 nm
Blue	0%	0%	8%	2 nm	3 nm	67 nm
Magenta	1.5%	0%	7.8%	9 nm	3 nm	44 nm
Cyan	0%	1.5%	8.3%	2 nm	20 nm	73 nm
White	1.3%	2.3%	5%	10 nm	48 nm	111 nm

Table S1. Diffraction efficiency in percentage observed in transmission for grating combinations for different colours. The power in the first diffraction order for each grating is normalized by the power transmitted through the sample in an unpatterned area. The individual colour SRGs were inscribed sequentially, starting with the red gratings, followed by green and blue. When multiple gratings are combined, the diffraction efficiency of the first grating decreases slightly. The grating heights were observed with the DHM after the patterning, and the different grating components were extracted with Fourier analysis similar to the Figure 5 of the main article.

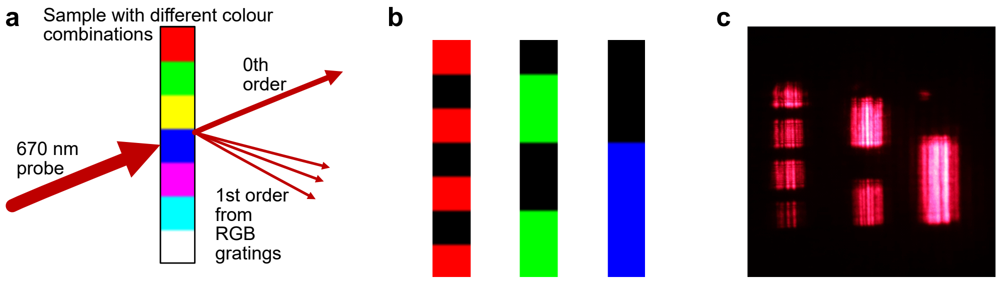

Figure S1. (a) Schematic of the diffraction efficiency measurement. A 670 nm probe laser hits a sample with areas patterned with gratings designed to diffract red, green, and blue light into the same angle in various combinations to realize different colours. Each of these grating components creates a diffraction spot for the 670 nm probe light, and the power in these spots is measured. (b) Distribution of the grating components in the differently coloured areas of the sample. The first column corresponds to the areas with red colour, the second to the green and third to blue. As the system is probed with a single wavelength, these areas show as separate regions under wide view illumination. (c) Photograph of the first diffracted orders under wide view illumination. From left to right, the first column shows the pixels with red colour, the second column the pixels with green colour and the third column the pixels with blue colour, matching the schematic in (b).

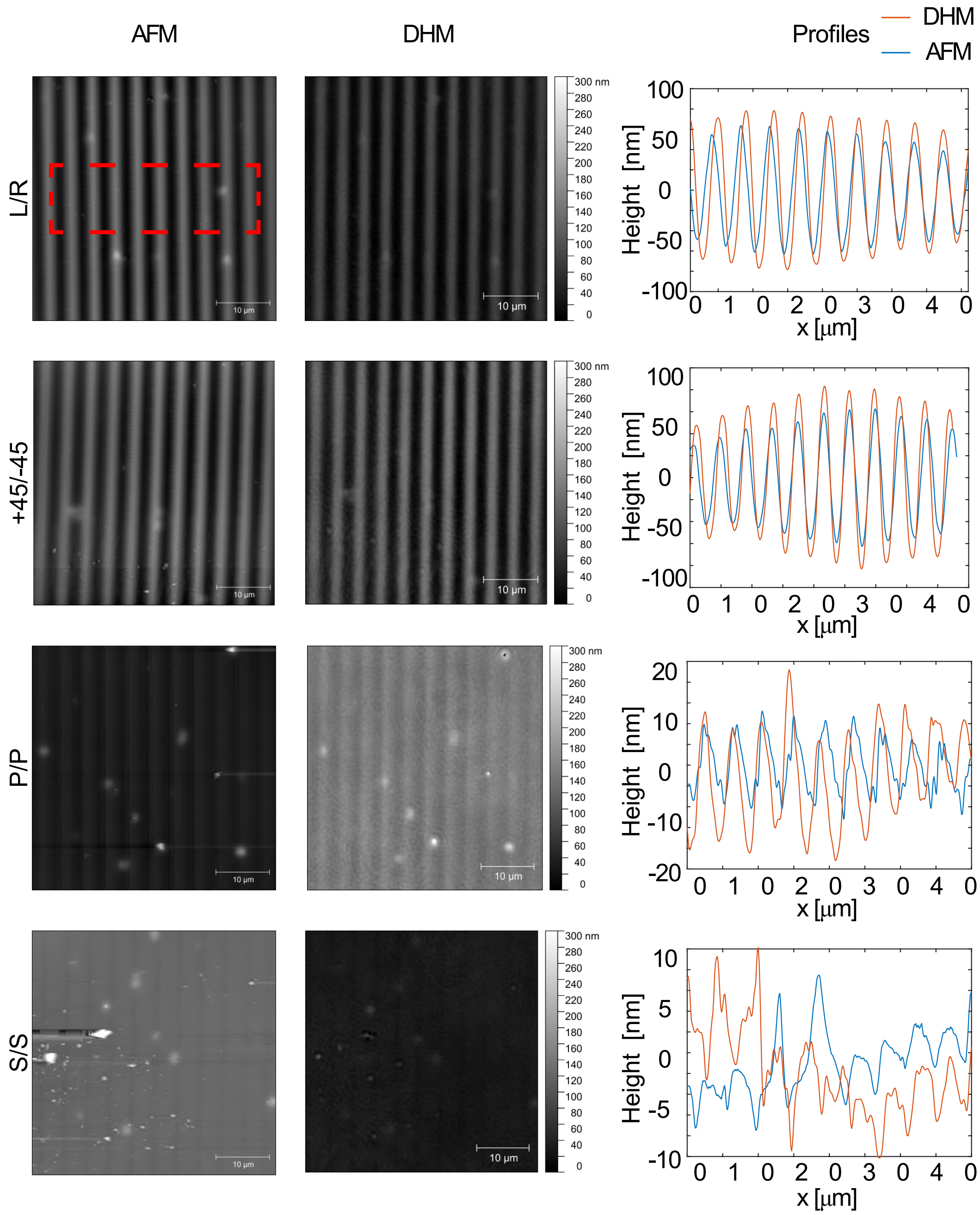

Figure S2. Comparison between gratings profiles inscribed with different polarization combinations, measured with DHM and AFM. The two measurement methods show a reasonable agreement between the gratings and all defects present on the sample. The AFM has a higher lateral resolution, and the defects on the sample allow us to match the measured areas between the two methods. The reported heights have some differences between them, which we attribute to the DR1g film being transparent at the DHM probe wavelengths. This leads to an additional contribution from reflections within the DR1g film, which is not taken into account in the current image reconstruction scheme.

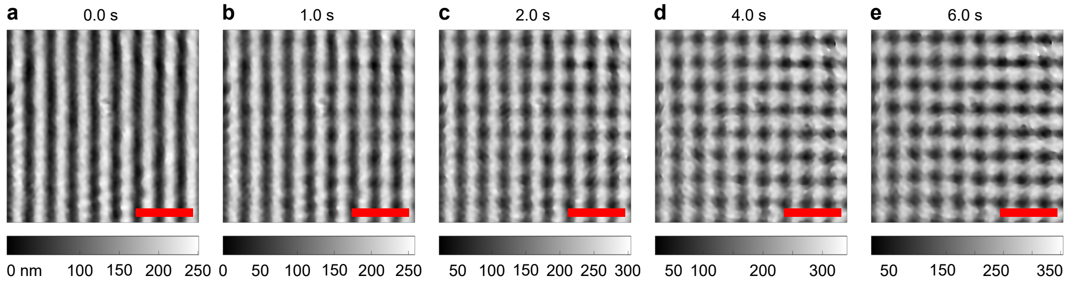

Figure S3. Grating growth during the second exposure for a 2D grating. First, a grating with 4 µm period was inscribed using circular polarizations (a). Then the illumination pattern was rotated 90 degrees, and the same location on the sample exposed for a second time. The panels (b) - (e) show the evolution of the grating height map as the 1D grating is transformed into a 2D grating structure. The scale bar corresponds to 10 µm.
